# Sexual and reproductive health of CDC plantation camp residents: a focus on unmet need for family planning among women in union

**DOI:** 10.1186/s12889-023-15114-y

**Published:** 2023-01-28

**Authors:** Agbor Nathan Emeh, Ngouakam Hermann, Elvis Asangbeng Tanue, Nsagha Shey Dickson

**Affiliations:** 1 Department of Public Health and Hygiene, Faculty of Health Sciences, University of Buea, P.O. Box 12, Buea, Cameroon; 2 Faculté de Médecine Et Des Sciences Pharmaceutiques, Université de Douala, B.P, 2701 Douala, Cameroun

**Keywords:** Sexual health, Reproductive health, Unmet need for family planning, Contraception, Women in union

## Abstract

**Background:**

Sexual and reproductive health is crucial to a normal and healthy female life. However, little interest has been placed on this subject particularly in the resource-limited settings of Cameroon. The study assessed the sexual and reproductive health of women in union, resident in the Cameroon Development Corporation (CDC) plantation camps, Cameroon.

**Methods:**

This was a cross-sectional study carried out from December 2019 to February 2020 in which a multi-stage sampling was applied in two purposively selected CDC plantation camps (Tiko and Penda Mboko). Out of the 16 clusters making up the camps, 8 were randomly selected using simple balloting. The main street junctions of the sampled clusters were identified and a direction of sampling randomly chosen. All houses left to the data collectors were sampled for eligible participants (one participant per household) and data were collected using validated interviewer-administered questionnaires. The number of participants per cluster was proportionate to population size of cluster. Data was analysed using SPSS 16 and statistical significance was set at *p* < 0.05. Regression analysis was used to determine predictors of unmet need for family planning.

**Results:**

Out of the 414 participants included, primary education was the highest level of education for a majority (43.0%). Most of the participants (44.7%) earned between 44.5–89.0USD/month. Relatively high proportions of some sexual and reproductive indicators like early sexual contacts (before 15 years) [87(21.0%)], grand multiparity [41(9.9%)], and abortion ≥ 3 [8(1.9%)] were recorded in the study. Two hundred and seventy-eight (278) participants (67.1%) [95%CI:62.4–71.7] used contraceptives and 90 (21.7%) [95%CI:17.9–26.0] had an unmet need for family planning with 3 major reasons for non-use of contraception among them being fear of side effects, discouragement from the partner, and lack of sufficient information on contraception. Of the different predictors of unmet need for family planning assessed, nulliparity/primiparity were protective for unmet need, and this was statistically significant (AOR = 0.284[0.086–0.934]).

**Conclusion:**

The sexual and reproductive health of CDC plantation camp residents is poor, and a health intervention is needed to improve it.

## Background

Reproductive health is a state of complete physical, mental and social well-being and not merely the absence of disease or infirmity in all matters relating to the reproductive system and its function and processes [1]. Although it may have a broad scope of indicators, few of them have beenwell-studied in most developing countries.

In most countries, the average age at first marriage is 18 years for females and 27 years for males, while rural females tend to marry even earlier [2]. Under Cameroon law, a girl must be at least 15 years old before she can get married [3]. Child marriage remains a major issue in Cameroon, where in 2017, over 10% and 31% of girls got married by 15 and 18 years, respectively [4]. According to UNICEF’s State of the World’s Children 2016, Cameroon ranks 20^th^ in the countries with the highest prevalence of child brides, and this is most common in the North of Cameroon, where 79% of girls marry early [5]. Consequently, adolescent fertility rate (births per 1000 women ages 15–19) in Cameroon stands out to be relatively high. The most recent statistical estimate in 2019 showed the adolescent fertility rate in Cameroon is about 106 births per 1000 women aged 15–19 [6]. A retrospective study (2009–2012) showed that over 13% of deliveries at the Buea Regional Hospital in Cameroon are teenage births [7]. The general fertility rate (births per woman) for Cameroon has experienced a steady decrease from 6.7 in 1985 to 4.6 in 2019, with projections of reaching 3.16 by 2050 [8].

The abortion rate represents another very important indicator of reproductive health. However, there is a general scarcity of literature on pregnancy loss in Cameroon. The present trend in publications shows a growing literature on this peril of pregnancy, most of which are anthropological studies [9]. According to a hospital-based study (2006–2010) carried out in Yaoundé, Cameroon, abortion is a leading cause of maternal deaths, accounting for over 25% of maternal deaths cases [10], the majority of which are induced/illegal abortions and therefore unsafe [11]. In a study carried out in an urban and a rural area in two different regions of Cameroon in 2011, it was reported that the prevalence of voluntary induced abortion was about 26.3% [12].

Family planning forms one of the ‘four pillars’ of Safe Motherhood [13] and has been well recognised for its impact on maternal morbi-mortality [14], infant outcomes [15], population growth [16] and poverty [17]. These health and population benefits of family planning make its access a human right as it protects the health of the mothers and children [18], and it has been stated as a single “best buy” in the achievement of all the 17 sustainable development goals (SDGs) [19]. Having adequate access to family planning and choosing when (if at all) to have children is a key measure of sustainability and development [20]. Family planning (FP) ensures that a woman only gets pregnant at a convenient time in her life when the pregnancy is wanted and planned. However, many women still do not use modern contraceptives, and as a result, the number of unintended pregnancies, abortions and, subsequently, deaths is high [15]. In less developed countries, contraceptive use continues to remain very low [21], and these are the countries with the highest maternal and infant mortalities. In 2017, more than 309 million women and girls were using modern contraception in 69 of the focus countries identified by Family Planning 2020 (FP2020), representing an increase of 38.8 million compared to figures from 2012 [20]. Although the target is far from being reached in most African countries, this shows that at least progress is being made towards meeting targets for contraceptive use as largest positive changes in modern contraception has been recorded in countries like Mozambique and Kenya [20].

Many women do not want to get pregnant or want to space their pregnancies, but they are not using contraceptives. Unmet need for family planning is a major public health problem, particularly in developing countries where it contributes to high maternal mortality levels [22]. Over 342,203 women died of maternal causes in 2008, and it was estimated that contraceptive use averted 272,040 maternal deaths (44%) [23]. According to Guttmacher Institute figures estimates, if all the unmet need for modern contraception were satisfied in developing countries, there would be three-quarters fewer unintended pregnancies, unplanned births and induced abortions per year, as well as 76,000 fewer maternal deaths [20]. Several studies have been carried out in different parts of the globe to assess the reasons and determinants of unmet need for family planning [24, 25]. The most common reasons for non-use of contraception amond women with unmet need for FP listed include fear of side effects and health risks associated with contraceptive use, infrequent sex, opposition of contraceptive use by others, misconception, lack of information and low perceived risk of pregnancy. However, these studies have shown contrasting determinants of having an unmet need for family planning. Generally, higher unmet need for family planning has been associated with women having more living children, women whose husbands desired more children than them, those ignoring their husband’s desired children [22], higher level of education, late marriage, poor knowledge of FP, poor male participation [25].

Analysis of Demographic Health Survey data (2012) has shown unmet need for family planning to have increased in Cameroon. There was a drastic decrease in the unmet need for family planning from 22.0 in 1991 to 9.6 in 1998, followed by a steady increase to 14.5 in 2004 and then to 16.6 in 2011 [26]. A study in Cameroon showed an unmet need for FP of 20.4% in an urban setting in Yaoundé [21] and an even higher prevalence of 46.6% in a rural setting in the North West [27]. According to DHS 2018, 23% of married women age 15–49 have an unmet need for family planning -15% for spacing and 8% for limiting [28].

We presume that unmet need for family planning will be much higher in the poorest settings of Cameroon. The low socioeconomic status of plantation camp labourers, in addition to the lack of an accessible family planning service within the CDC plantation camps, may expose them to some adverse sexual and reproductive health risks. In addition, the present socio-political crisis in the Anglophone zone have negatively affected the economic status of residents leaving many CDC camp labourers unpaid. This study aimed to assess the sexual and reproductive health profile of women in union aged 15–49 years resident in the Cameroon Development Corporation plantation camps and identify the determinants of unmet need for family planning in the poor cohort setting (CDC plantation Camps). So far, we are not aware of any study carried out to assess the sexual and reproductive health predictors of residents in the CDC plantation camps. The result of this study can help to design public health interventions that may be helpful to improve the sexual and reproductive health of this population.

### Definition of operational terms

Sexual and reproductive health: This is a state of complete physical, mental and social well-being in all aspects patterning to the reproductive system, thereby permitting them to have a satisfying and safe sex life, ability to reproduce, and the freedom to decide if, when and how often to do so [29].

**Fig.1:**
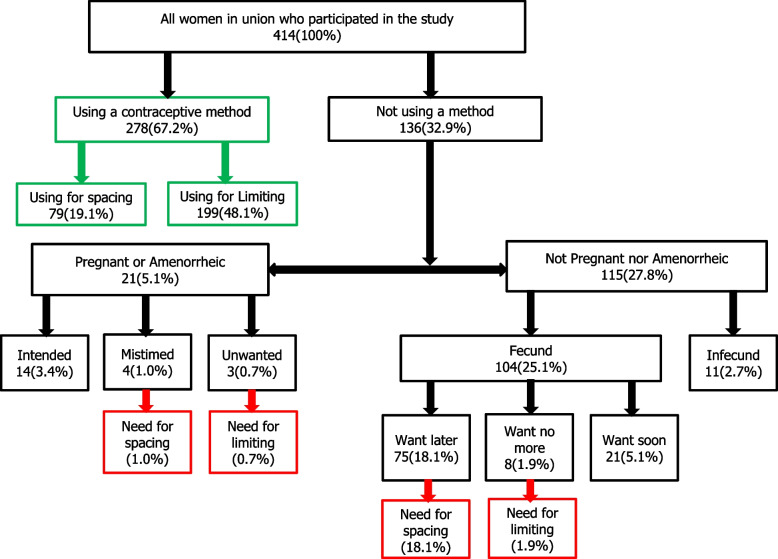
Unmet need for family planning of women in union (15–49 years) in CDC plantation camps

Unmet need for family planning: A woman has unmet need for family planning if she is fecund and sexually active and desires to either end (limiting) or postpone childbearing for at least two years (spacing), but is not currently using a contraceptive method [30]. Those who had a present pregnancy or their last pregnancy unplanned were also included as having unmet need for family planning. This classified them into two groups of women as shown in Fig. 1: (a) those with an unmet need for limiting and (b) those with an unmet need for spacing. Unmet need for family planning was therefore calculated as (a) + (b).

Contraceptive user: Any woman in a union currently doing something or using any method to delay or avoid getting pregnant.

Fecund: A woman is said to be fecund if she can get pregnant. In this study, we defined a woman as being fecund if she was either pregnant at the time of study or had at least one pregnancy in the past.

Woman in union: We considered a woman as being in a union when she is living under the same roof with a man, either in a free (or consensual) union or legal marriage.

Early sexual contact: We defined a participant as having early sexual contact if sheattested to having had her first sexual relationship with a male partner before the age of 15 years.

## Methods

### Study area and setting

The study was conducted in the CDC plantation camp. These sites were purposively determined as intervention and control sites for a clustered non-randomized controlled trial that aimed at assessing the impact of family planning intervention on maternal and infant mortality in the Tiko CDC plantation camp, a high maternal mortality zone. The CDC plantation that has existed since 1947 has several camps all over the country, with over 22,409 employees living within the camps [31]. Among the largest and well-known are Camp Tilo, Camp Nkoume and the SOCAPALM in Nkappa, in the Dibombari Health District in Littoral region. However, the study was conducted in the CDC camps settings of Tiko (in the South West region) and Penda Mboko (in the Littoral Region), purposively selected for their accessibility and representativity. Tiko is bounded by Limbe, Buea, Muyuka, Dibombari and Bonaberi to the West, North, North-East, East and South, respectively and has a total surface area of 4,840 km2. Tiko CDC camp has geographical coordinates 4º 4′ 30″ North and 9º 22′ 0″ East, and the Penda Mboko camp coordinates 4º 16′ 30″ North and 9º 26′ 30″ East. The camps consist of clusters of settlements constructed to harbour CDC plantation workers, for which there are over 600 houses in the Tiko camp and 450 in the Penda Mboko camp. A typical labourer works 5 working days a week from 7 am to 6 pm with minimal wages. They also benefit from a free health dispensary within the camps, which provides first-line health management to the labourers. However, this units do not have any functional family planning services.

### Study design

The study was a community-based cross-sectional study in which participants were enrolled in their settlements in the CDC plantation camps of Tiko and Penda Mboko. The study was conducted from December 2019 to February 2020.

### Selection criteria

Women of reproductive age group (15–49 years) in union, who had been resident in the Tiko or Penda Mboko CDC plantation Camps for at least 2 years prior to the study and provided informed consent/assent for participation in the study. Those who did not fulfil these requirements were not included.

### Sample size

We calculated the minimum sample size using Cochran’s formula [32].

$$\begin{array}{c}\mathrm{n}={\mathrm{Z}}^{2}\mathrm{P}\left(1-\mathrm{P}\right)\left(\mathrm{Deff}\right),\\ {\mathrm{d}}^{2}\end{array}$$

where Z = 1.96 at a 95% confidence interval, from a previous survey in Cameroon, we estimate the unmet need for family planning (P) = 20% = 0.20 (estimated unmet need for family planning in Cameroon) [21], d = 5% = 0.05 (error margin), Design effect (Deff) = 1.1.

$$\begin{array}{cc}\mathrm{n}= {\mathrm{Z}}^{2}\mathrm{P}\left(1-\mathrm{P}\right)\left(\mathrm{Deff}\right)=& {\left(1.96\right)}^{2}\left(0.20\right)\left(0.80\right)\left(1.1\right)=270\\ {\mathrm{d}}^{2}& {\left(0.05\right)}^{2}\end{array}$$

However, a total sample of 414 women in union of reproductive age was used in the study because we had available resources and desired to work at a higher power with a better precision. The selection of the participants was based on probability proportionate to size.

### Sampling technique

Two CDC plantation camps (Tiko and Penda Mboko) located in two different regions (South West and Littoral regions, respectively) were purposively selected. Each of these camps was divided into clusters which represented their pre-existing camp quarters. Tiko had nine (9) clusters from which we randomly selected four (4) clusters using a simple ballot. Likewise, Penda Mboko had seven (7) clusters from which we randomly selected four (4) using the same method.

The main street junctions of each cluster were identified, and then a direction of household data collection within each cluster was randomly selected by rotating a plastic bottle. The direction of the head of the bottle indicated the direction of data collection. At the start of this main junction and moving in the selected direction, data collectors systematically visited all houses situated on their left-hand side. At the household level, only one eligible participant was selected; this was the first eligible participant encountered by the data collector. The number of participants per cluster was selected using probability proportionate to size.

### Data collection procedures

#### Administrative clearance

Administrative clearances were obtained from the Regional Delegation of Health of the South West Region and Littoral Region. A written authorization was obtained from the Director of Human Resources of the CDC.

#### Ethical considerations

The study was approved by the Institutional Ethics Committee for Research on Human Health of the University of Douala (Ref.:2069 IEC¬ UD/12/2019/T), informed consent/assent was obtained from all subjects and/or their legal guardian(s). All experiment protocol for involving humans was in accordance with national/international/institutional guidelines.

#### Pretesting

Twenty (20) questionnaires were pretested in one of the CDC plantation camps clusters to ensure that the questions were well understandable and had the same meaning to both the data collectors and potential participants. After the pretesting, some adjustments were made to the questionnaire, and it was validated after review by research experts in the field of study. Major modifications made included reformulating some questions and elimination of question of gender since all the participants were women. Pretested questionnaires were not included in the data analysis.

#### Training of data collectors

One week prior to data collection, a three days training seminar for the data collectors led by the principal investigator was done. The data collectors for this study had a minimum academic qualification of Bachelor’s degree in a biomedical science field and a working experience of at least two previous community research data collection. Adult learning techniques were used in training, including presentations, questions and answers, brainstorming and role play. The training seminar had three modules, one for each day; module 01 covered the importance of family planning and the study objectives, module 02 covered the details of the structured questionnaire and module 03 covered community entry procedures, how to carry out the fieldwork and anticipated challenges.

#### Field work

After collecting the ethical clearance and administrative authorizations, community chiefs and quarter heads were visited, from whom authorization to access the communities was obtained. Access into the communities was led and guided by community health workers (CHWs) of the Tiko and Penda Mboko health districts in their respective districts. Trained data collectors collected data in the two districts simultaneously throughout the study. Surveyors visited each sampled household and inquired whether or not there was a potential participant (women in union) in the household. In households where participants were presently on sit, data were collected; otherwise, surveyors revisited the sampled household as many times as possible until the eligible participant (if present) was met and interviewed. Only one participant was selected per household, and this was the first eligible participant encountered in that home. In cases where the first contacted eligible participant in a particular household refused to participate, subsequent eligible participants of the same household were contacted. After providing informed consent/assent to participate in the study, they were required to answer the interviewer-administered study questionnaire on a one-to-one basis in a quiet location away from the hearing of the other household members.

Data were captured using a pretested interviewer-administered semi-structured questionnaire. The questionnaire covered socio-demographic information of the study participants, including age, marital status, education and income level. The questionnaire also covered reproductive health indicators and a history of contraception.

#### Data management and analysis

Filled questionnaires were checked by the principal investigator, and those that lacked important information (identification number, age, etc.) were excluded. Data were entered into the EpiData version 3.1 data entry form created to contain checks so as to avoid entry errors. The database was then cleaned in the same software, and exported and analysed in SPSS version 16. Frequencies were calculated for categorical variables, while means were used to describe continuous variables. Unmet need for family planning was calculated using the Westoff/Demographic and Health Survey method [33]. See Fig. 1. Odd ratio (and 95% confidence interval) was used to measure the strength of association between unmet need for family planning and the different covariates selected based on previous studies in this field. Multivariate analysis was done by introducing all covariates for which simple logistic regression analysis showed statistically significant association with unmet need into the multiple logistic regression model. Statistical significance was considered at *p*-value < 0.05 for both the simple and multiple logistic regression.

## Results

### Demographic characteristics

Table 1 shows the demographic characteristics of the study population. Out of 438 eligible participants who were contacted for the study, 24 (5.5%) refused to participate with their main reason that they needed financial benefit for their participation. A total of 414 women were included in the final sample of this study, with their ages ranging from 15–49 years (mean = 33.0 ± 7.137). The majority of the participants were those of age ranging from 25–35 years (192; 46.4%); 335 (80.9%) were in a legal union; 178 (43%) had primary education as their highest level of studies, and this represented the majority of the participants; 281 (67.9%) were Pentecostals, and a majority (44.7%) earned between 44.5–89.0USD per month.

**Table 1:** Socio-demographic characteristics of women in union aged 15–49 in CDC plantation camps

Variables	Tiko №(%)	Penda Mboko №(%)	Total №(%)	Ҳ^2^(Df)	*p*-value
Age Range (years)					
< 25	41(19.6)	29(14.1)	70(16.9)	12.49(2)	0.002*
25–35	79(37.8)	113(55.1)	192(46.4)		
> 35	89(42.6)	63(30.7)	152(36.7)		
Total	209(50.5)	205(49.5)	414(100)		
Marital Status					
Married	167(79.9)	168(82.0)	335(80.9)	0.28(2)	0.596
Consensual union	42(20.1)	37(18.0)	79(19.1)		
Total	209(50.5)	205(49.5)	414(100)		
Education Level					
None	58(27.8)	74(36.1)	132(31.9)	3.82(3)	0.282
Primary	96(45.9)	82(40.0)	178(43.0)		
Secondary	30(14.4)	30(14.6)	60(14.5)		
Tertiary	25(12.0)	19(9.3)	44(10.6)		
Total	209(50.5)	205(49.5)	414(100)		
Religion					
Christians	204(97.6)	201(98.0)	405(97.8)	2.13(2)	0.345
Muslim	3(1.4)	4(2.0)	7(1.7)		
None	2(1.0)	0(0)	2(0.5)		
Total	209(50.5)	205(49.5)	414(100)		
Income Level (USD)					
< 44.5	48(23.0)	41(20.0)	89(21.5)	1.97(3)	0.579
44.5–89.0	94(45.0)	91(44.4)	185(44.7)		
89.0–133.5	47(22.5)	57(27.8)	104(25.1)		
> 133.5	20(9.6)	16(7.8)	36(8.7)		
Total	209(50.5)	205(49.5)	414(100)		

Ҳ^2^ = chi-square

*Df*  Degree of freedom

^*^ = statistically significant

### Sexual and reproductive health profile

Table 2 summarises the sexual and reproductive health profile of CDC plantation camp residents. The ranges and means of important sexual and reproductive indicators assessed in this study include the age at which participants got into a union (range = 8–42 years; mean = 21.61 ± 5.382), age at which participants had their first sexual contact (range = 8–28 years; mean = 17.25 ± 2.288), gravidity (range = 0–9; mean = 3.17 ± 1.798), number of abortions or miscarriages ever experienced (range = 0–6; mean = 0.59 ± 0.934), number of participants’ children who were alive at the time of the study (range = 0–8; mean = 2.56 ± 1.570), number of children who were below 2 years of age at the time of the study (range = 0–3; mean = 0.34 ± 0.562). Other reproductive parameters assessed in the study included the duration of the last pregnancy, the outcome of the last pregnancy, fertility status and present pregnancy status.

**Table 2:** Sexual and reproductive health profile of women in union aged 15–49 in CDC plantation camps

Variables	Tiko №(%)	Penda Mboko №(%)	Total №(%)	Ҳ^2^(Df)	*p*-value
Age of getting into union					
< 25	163(78.0)	162(79.0)	325(78.5)	0.98(2)	0.614
25–35	43(20.6)	42(20.5)	85(20.5)		
> 35	3(1.4)	1(0.5)	4(1.0)		
Total	209(50.5)	205(49.5)	414(100)		
Age of first sexual contact					
< 15 years	44(21.1)	43(21.0)	87(21.0)	0.82(3)	0.845
15-20 years	154(73.7)	149(72.7)	303(73.2)		
20-25 years	9(4.3)	12(5.9)	21(5.1)		
> 25 years	2(1.0)	1(0.5)	3(0.7)		
Total	209(50.5)	205(49.5)	414(100)		
Number of pregnancies ever had in life					
Nulliparous	15(7.2)	12(5.9)	27(6.5)	1.04(3)	0.792
Primiparous	26(12.4)	24(11.7)	50(12.1)		
Multiparous	150(71.8)	146(71.2)	296(71.5)		
Grand Multiparous	18(8.6)	23(11.2)	41(9.9)		
Total	209(50.5)	205(49.5)	414(100)		
Number of abortions/miscarriages					
0	135(64.6)	125(61.0)	260(62.8)	5.48(3)	0.140
1	49(23.4)	42(20.5)	91(22.0)		
2–3	20(9.6)	35(17.1)	55(13.3)		
> 3	5(2.4)	3(1.5)	8(1.9)		
Total	209(50.5)	205(49.5)	414(100)		
Number of children alive					
0	21(10.0)	21(10.2)	42(10.1)	0.05(2)	0.976
1–3	132(63.2)	131(63.9)	263(63.5)		
> 3	56(26.8)	53(25.9)	109(26.3)		
Total	209(50.5)	205(49.5)	414(100)		
Number of children 2 years and below					
0	144(68.9)	145(70.7)	289(69.8)	2.36(2)	0.307
1–2	65(31.1)	58(28.3)	123(29.7)		
> 2	0(0)	2(1.0)	2(0.5)		
Total	209(50.5)	205(49.5)	414(100)		
Time of last pregnancy					
< 3 years ago	86(44.6)	100(51.5)	186(48.1)	5.54(2)	0.063
3–6 years ago	47(24.4)	54(27.8)	101(26.1)		
> 6 years ago	60(31.1)	40(20.6)	100(25.8)		
Total	193(49.9)	194(50.1)	387(100)		
Outcome of last pregnancy					
Singleton	178(92.2)	176(90.7)	354(91.5)	0.44(2)	0.804
Multiple	6(3.1)	6(3.1)	12(3.1)		
Miscarriage/abortion/child death	9(4.7)	12(6.2)	21(5.4)		
Total	193(49.9)	194(50.1)	387(100)		
Fecundity status					
Fertile	203(97.1)	200(97.6)	403(97.3)	0.07(1)	0.785
Infertile	6(2.9)	5(2.4)	11(2.7)		
Total	209(50.5)	205(49.5)	414(100)		
Present pregnancy status					
Pregnant	9(4.3)	12(5.9)	21(5.1)	0.52(1)	0.473
Not pregnant	200(95.7)	193(94.1)	393(94.9)		
Total	209(50.5)	205(49.5)	414(100)		

### Unmet need for family planning, reasons for non-use of contraception among women with unmet need

Figure 1 summarises contraceptive prevalence, unmet need for family planning and other related concepts in the CDC plantation camps. While over 278 (67.2%) [95%CI:62.4–71.7] of women used contraceptive methods at the time of the study, a majority (199; 48.1%) [95%CI:43.2–53.0] used contraceptives for limiting pregnancy.

Infertility rate in the CDC plantation camps stood at 2.7% [95%CI:1.3–4.7]. Ninety (90) participants (21.7%) [95%CI:17.9–26.0] had unmet need for family planning; 79 (19.1%) [95%CI:15.4–23.2] for spacing and 11 (2.7%) [95%CI:1.3–4.7] for limiting pregnancy. As seen in Table 3, major reasons for non-use of contraception among women with unmet need for family planning included fear of side effects (51.1%), opposition from partner (30.0%), lack of sufficient information on contraceptive methods (13.3%) and not seeing contraceptive as being necessary (10.0%). The potential demand for contraception, which is the percentage of current contraceptive users added to the percentage of women with unmet need for family planning (excluding the women with unmet need who were either pregnant or amenorrheic) stood at 87.2% [95%CI:83.6–90.3]: 37.2% [95%CI:32.5–42.1] for spacing and 50.0% [95%CI:45.1–54.9] for limiting. Only 77.0% (278/361) [95%CI:72.3–81.3] of the overall potential contraceptive demand was satisfied at the time of this study.

**Table 3:** Reasons for non-use of contraceptives among women with unmet need for family planning

Reasons	Yes №(%)	No №(%)	Total №(%)
Fear of side effects	46(51.1)	44(48.9)	90(100)
My health personnel told me not to	1(1.1)	89(98.9)	90(100)
My partner stopped me from using it	27(30.0)	63(70.0)	90(100)
A family member discouraged me	1(1.1)	89(98.9)	90(100)
Someone discouraged me	2(2.2)	88(97.8)	90(100)
Could no longer access family planning	2(2.2)	88(97.8)	90(100)
Not having regular sex	6(6.7)	84(93.3)	90(100)
Need to know more concerning contraceptive methods	12(13.3)	78(86.7)	90(100)
Contraceptives causes sexual dissatisfaction	3(3.3)	87(96.7)	90(100)
I don’t see contraceptive as necessary for me	9(10.0)	81(90.0)	90(100)
I don’t know	15(16.7)	75(83.3)	90(100)

### Predictors of unmet need for family planning

The predictors of unmet need for family planning assessed in the study included participants’ age, marital status, level of education, level of income, site of residence, age at which participants got into union, age of first sexual contact, gravidity, number of children who were presently alive, the outcome of last pregnancy, number of abortions/miscarriages and fecundity. The Table 4 shows the determinants of unmet need for family planning in the CDC plantation camps.

**Table 4:** Determinants of unmet need for family planning in the CDC plantation Camps

Variables	Unadjusted OR [95%CI]	*p*-value	Adjusted OR [95%CI]	*p*-value
Participant having age < 35 years (Y/N)	1.179[0.569–2.445]	0.657	0.983[0.465–2.077]	0.964
Married (Y/N)	0.612[0.246–1.520]	0.290	1.497[0.590–3.797]	0.396
Level of education below Primary (Y/N)	1.143[0.471–2.775]	0.768	0.754[0.293–1.939]	0.558
Christian (Y/N)	7.286[1.508–35.211]	0.013*	1.255[0.595–2.649]	0.551
Having income level < 89.0USD (Y/N)	0.783[0.358–1.711]	0.539	1.406[0.632–3.129]	0.403
Living in Tiko CDC Camp (Y/N)	0.661[0.417–1.050]	0.080	0.571[0.266–1.223]	0.149
Age of getting into union < 30 years (Y/N)	0.395[0.139–1.120]	0.081	2.588[0.909–7.369]	0.075
First Sexual Contact age < 20 years (Y/N)	1.935[0.442–8.458]	0.381	0.413[0.086–1.991]	0.270
Nulli/primiparous (Y/N)	3.368[1.035–10.965]	0.044*	0.284[0.086–0.934]	0.038*
Having < 3 Children (Y/N)	1.257[0.585–2.698]	0.558	0.951[0.430–2.100]	0.901
Last pregnancy outcome: Singleton (Y/N)	0.941[0.319–2.774]	0.912	1.546[0.686–3.484]	0.293
Last pregnancy: < 5 years ago (Y/N)	0.563[0.247–1.281]	0.171	1.844[0.800–4.251]	1.151
Number of abortions: < 3 Abortions (Y/N)	0.474[0.104–2.157]	0.334	1.657[0.264–10.392]	0.590
Fecund (Y/N)	2.769[0.280–27.352]	0.383	1.330[0.093–19.122]	0.834

*Y/N*  Yes/No, *OR* Odds Ratio, *Adjusted OR* Adjusted Odds Ratio, *CI* Confidence Interval

^*^ = Statistically significant (*p*-value ≤ 0.05)

When the odds were unadjusted, statistically significant predictors of unmet need for family planning included being a Pentecostal (OR = 7.286[1.508–35.211], *p* = 0.013) and nulliparous/primiparous (OR = 3.368[1.035–10.965], *p* = 0.044). However, when adjusted in a multiple logistic regression model, only gravidity status was a statistically significant predictor of unmet need for family planning; being nulliparous/primiparous (AOR = 0.284[0.086–0.934], *p* = 0.038).

## Discussion

This was a community-based cross-sectional study that assessed the sexual and reproductive health profile of women in union aged 15–49 years resident in the CDC plantation camps, focusing on their unmet need.

As seen in Table 1, the middle age group (25–35 years) constituted the majority of the reproductive women.

Over 21% [95%CI:17.2–25.3] of the study participants had their first sexual contact at an age less than 15 years, with the earliest age at first sexual contact being 8 years. Recent studies have confirmed high sexuality among Cameroonian adolescents [29]. This high rate of teenage sexual activity is the root cause of the high teenage pregnancy in Cameroon, which is, on its own, a major public health issue [34]. Grand multiparity in the study area stood at 9.9% at the time of the study, meaning about 1 in every 10 women has had at least 5 births. An even higher prevalence of grand multiparity has been reported in some rural communities in Cameroon [35]. Grand multiparity has remains an obstetric risk because of the documented complications associated with the condition [36]. Other sexual and reproductive health indicators assessed in this survey included the number of abortions, timing of last pregnancy, outcome of the previous pregnancy and fertility status.

The unmet need for family planning in the present study was 21.7% (19.1% for spacing and 2.6% for limiting), which is about 1.3% higher than that reported by Ajong et al*.* in 2016 [21]. This means that over 1 out of every 5 women have an unmet need for family planning, which is relatively high. Even higher prevalence of unmet need for family planning has been registered in other parts of Cameroon [26]. In the North West region, for example, about 5 in every 10 women have an unmet need for family planning [27]. These differences in unmet need for family planning could be explained by the context-specific discrepancies. Access to information on contraception, access to contraceptive methods and availability of qualified and trained personnel varies within these settings. The study conducted in the North West Region was carried out in a rural area, usually characterized by reduced availability of reproductive health services.. Secondly, earlier studies had reported relatively higher and even an increasing trend of unmet need for family planning in rural settings compared to urban settings [26]. Unmet need for family planning has to be kept at its lowest rate if family planning targets for the post-2015 sustainable development goals are to be achieved. This indicates that more interventions have to be carried out at the local and national levels to increase uptake of family planning, particularly in rural settings. The prevalence of contraceptive use at the time of this study stood at 67.1% (19% for spacing and 48.1% for limiting). The finding that about 10 in every 15 women use contraceptive is quite encouraging and also an indication of positive progress towards achievement of the post-2015 targets of universal access to family planning [27]. While this prevalence is over 5 × greater than that reported by Edietah et al*.* in the North West Region of Cameroon in 2018 [27], two reasons could account for this. Firstly, in the present study, the reported contraceptive prevalence takes into consideration both modern and traditional methods as means of controlling pregnancies while the previous study considered only modern methods. This appears to be a limitation in the present study as some do not consider the traditional contraceptive methods as an effective way to control pregnancy due to their relatively low protection [37]. However, no matter how one may desire to use family planning, a woman can only use the method that is available at her disposal and which she feels she best understands. In a resource-limited setting like the CDC plantation camps where a majority of the residents earn below 89.0USD per month, and where availability of modern contraceptive methods cannot be guaranteed on a regular basis, contraceptive users are more likely to rely on the less expensive and readily available traditional methods which they think they best understand, the rhythm method as in this case. Secondly, contraceptive prevalence has been reported to increase over time as several strategies, including education, are continually being put in place in an attempt to achieve universal access to contraception, an SDG 5 target [27].

A potential demand for contraception of 87.2% of the target population shows a large proportion of the population who can actually benefit from targeted and effective family planning services. However, only 77.0% of this demand was satisfied, indicating that over 23% of the target population who should have benefited from contraception do not use it. Although it was not the focus of this paper to evaluate the proportion of demands met by modern and traditional contraception, a contraceptive user in this study was defined as any woman in a union currently doing something or using any method as a means to delay or avoid getting pregnant. Therefore, with 77% of this demand being met in a rural setting such as CDC plantation camps, a majority of this satisfied demand is likely to come from the use of traditional method. The relatively low degree of efficacy of these traditional methods suggests to us that the contribution of this met demand in the improvement of reproductive health in the setting may be limited. While this high potential demand mirrors that of earlier studies in Cameroon [21], potential contraceptive demand as low as 45.9% was reported in the North West Region of Cameroon [27]. As will be expected, where the overall potential contraceptive demand is low, fertility often stands out to be high since fewer women will be using contraceptive methods, as in the North West Region.

The major reasons for non-use of contraception among women with unmet need for family planning in the CDC plantation camps included fear of side effects, opposition from partner and lack of sufficient information on contraceptive methods. This represents the reasons why women do not use contraception despite the fact that they actually want to avoid pregnancy (number of potential clients) and the reasons generally vary base on the study population characteristics [26]. This high level of unmet need are sometimes interpreted as evidence of a lack of access to contraceptive supplies and services in developing countries [38]. Earlier studies have reported fear of side effects, opposition from partners and not having frequent sex as major reasons for not using contraception by those with unmet need for family planning [21, 38]. In a more recent study in a rural community, major reasons for unmet need for family planning included medical reasons, fear of side effects and lack of information [39].

Several studies have assessed different predictors of unmet need for family planning, and their findings have been unique and, at times conflicting [21, 24, 28]. Among the several predictors of unmet need assessed in the present study, only religion and gravidity status were statistically significant following simple logistic regression. Being a Christian increased the odds of unmet need for family planning by over 7 folds compared to those attending other religious denominations. In the same light, nulliparous/primipa women had their odds for unmet need increased by 3 folds compared to multiparous/grand multiparous women. However, when adjusted, parity status was the single most important predictor of unmet need for family planning such that nulliparous/primiparous women had lower odds for unmet need for family planning than multiparous women. This is reasonable in that nulliparous and primiparous women are less likely to adopt FP in a union than multiparous women. In a study carried out in 2019 by Karol and colleagues in a rural community, factors significantly associated with unmet need for family planning included mother’s age, education, contraceptive knowledge, desired family size, age at first child birth and male participation [39]. In another analysis of the trend of unmet need for family planning in Cameroon showed that women with secondary or higher education have a lower unmet need for family planning than women with primary or no education [40]. Unmet need places a woman at higher risk of unwanted pregnancy. In a study carried out in Angola, it was shown that compared to women who had no unmet need, those with unmet need had their odds for experiencing unwanted pregnancy increased by 4–7 folds [41].

### Limitations and strength of the study

In this study, everyone using contraception (whether traditional or modern method) was considered to have a met need for family planning. Some literature has pointed out those using traditional methods of contraception as having an unmet need for family planning [42]. This is due to the relatively low effectiveness of the traditional methods in preventing pregnancy. However, in resource-limited settings like the CDC plantation camps where access to modern contraceptive methods is low and where healthcare workers do not master the handling of modern methods, settlers often tend to depend on traditional methods, which are readily available and easy to handle thus making the traditional methods a very useful form of contraception in these kind of settings [43]. The study did not evaluate the contribution of men and of contraceptive supply and delivery to unmet need for family. Also, sexually active women who were not in a union were not included since the study had to take into consideration other variables which could not be provided by women who were not in union. Participants had to confirm they were sexually active before inclusion in the study. Due to the sensitivity of this subject, we worked on the assumption that women who were living in union are sexually active. Cross-sectional design, as used in this study, is ideal for the evaluation of prevalence and association between variables of interest. However, it does not reveal causal relationship. Lastly, our definition of “fecund” as women who were either pregnant at the time of the study or had at least one child in the past will underestimate this variable since it excludes newly married women who have not yet gotten pregnant. The strength of the study is the fact that the two CDC plantation camps we used for the study not only were they located in two different regions of the country but are among the largest CDC camps in Cameroon. This implies that the findings of this study could be representation of the sexual and reproductive health of the CDC plantation camps in Cameroon.

## Conclusion

From this study, it was noticed that the indicators of sexual and reproductive health in the CDC plantation camps, such as sexual contact before 15 years, grand multiparity, multiple abortions and unmet need for family planning, were poor. The main reasons for the non-use of contraception among those with unmet need for family planning were fear of side effects and disapproval from partners, while the determinant for unmet need was the parity status. An oriented intervention comprising health education (to improve knowledge and counteract misconceptions) and increasing accessibility and availability of modern contraceptive methods in the CDC plantation camps may improve the sexual and reproductive health of these cohorts.

We recommend that future studies should assess the unmet need for family planning in men, the couple’s unmet need and unmet contraceptive needs among sexually active single women.

## Data Availability

All data generated or analysed during this study are included in this published article. Raw data can be access online on figshare (https://doi.org/10.6084/m9.figshare.11720619.v1).

## Abbreviations

CDC: Cameroon Development Corporation
CI: Confidence Interval
AOR: Adjusted Odd Ratio
SDG: Sustainable Development Goal
FP: Family Planning
